# Use of Urea for the Syndrome of Inappropriate Secretion of Antidiuretic Hormone: A Systematic Review

**DOI:** 10.1001/jamanetworkopen.2023.40313

**Published:** 2023-10-30

**Authors:** Ralph Wendt, Andrew Z. Fenves, Benjamin P. Geisler

**Affiliations:** 1 Department of Nephrology, St Georg Hospital, Leipzig, Germany; 2 Department of Medicine, Massachusetts General Hospital/Harvard Medical School, Boston

## Abstract

**Importance:**

Hyponatremia and the syndrome of inappropriate secretion of antidiuretic hormone (SIADH) are associated with significant mortality and morbidity. The effectiveness and safety of oral urea for SIADH are still debated.

**Objective:**

To evaluate the efficacy and safety of urea for the treatment of SIADH.

**Evidence Review:**

A systematic search of Medline and Embase was conducted for controlled and uncontrolled studies of urea for SIADH in adult patients. The primary outcome was serum sodium concentration after treatment. Secondary outcomes included the proportion of patients with osmotic demyelination syndrome (ODS), intracranial pressure, and resource use such as length of stay.

**Findings:**

Twenty-three studies involving 537 patients with SIADH were included, of which 462 were treated with urea. The pooled mean baseline serum sodium was 125.0 mmol/L (95% CI, 122.6-127.5 mmol/L). The median treatment duration with oral urea was 5 days. Urea increased serum sodium concentration by a mean of 9.6 mmol/L (95% CI, 7.5-11.7 mmol/L). The mean increase in serum sodium after 24 hours was 4.9 mmol/L (95% CI, 0.5-9.3 mmol/L). Adverse events were few, mainly consisting of distaste or dysgeusia, and no case of ODS was reported. Resource use was too infrequently reported to be synthesized.

**Conclusions and Relevance:**

In this systematic review of the use of urea in SIADH and despite the lack of randomized clinical trials, lower-quality evidence was identified that suggests that urea may be an effective, safe, and inexpensive treatment modality that warrants further exploration.

## Key Points

**Question:**

Is urea an effective, safe, and inexpensive treatment for patients with the syndrome of inappropriate secretion of antidiuretic hormone (SIADH)?

**Findings:**

This systematic review of 23 studies involving 537 patients with SIADH, of which 462 were treated with urea, found that while there is no definitive evidence from randomized clinical trials, the available lower-quality evidence suggests that urea may be an effective treatment option. Urea was found to increase serum sodium levels with a very low risk of overcorrection and infrequently reported adverse effects, primarily dysgeusia (distorted sense of taste).

**Meaning:**

These results suggest that, while definitive evidence from randomized controlled trials is currently lacking, data from previous studies support the hypothesis that urea might be an effective, safe, and inexpensive treatment option for patients with SIADH.

## Teaser

This systematic review assesses whether urea is an effective, safe, and inexpensive treatment option for syndrome of inappropriate secretion of antidiuretic hormone.

## Introduction

Hyponatremia, a common electrolyte disorder among hospitalized patients, is associated with increased mortality.^1^ Depending on the serum sodium level and the acuity, clinical presentations may include gait disturbance or various degrees of neurocognitive impairments.^2^ A common etiology for hyponatremia is the syndrome of inappropriate secretion of antidiuretic hormone (SIADH).^3^ The therapy for patients with hyponatremia due to SIADH depends on the severity of the hyponatremia and the clinical condition of the patient.

Although urea is not commonly used for SIADH treatment worldwide, it was recommended for this purpose in a 2014 joint guideline on diagnosis and treatment of hyponatremia by the European Renal Association–European Dialysis and Transplant Association, the European Society of Intensive Care Medicine, and the European Society of Endocrinology.^4^ However, this recommendation was classified as weak and was based on the lowest level of evidence (ie, grade D).

We therefore aimed to systematically review the currently available evidence for or against urea for hyponatremia from SIADH. Specifically, our objectives were to systematically review the effectiveness of urea vs vaptan receptor antagonists (VRAs) in terms of increasing the plasma sodium concentration of patients with SIADH, the safety of urea vs all other treatment regarding the incidence of osmotic demyelination syndrome (ODS), the cost of illness of SIADH and specifically the in-hospital resource utilization including length of stay, intensive care unit usage, specialist consultations, number or frequency of blood draws, and cost of prescription drugs. Additionally, we sought to compare the effectiveness of urea against treatments other than VRAs, including supportive measures such as treating the underlying disease, fluid restriction, sodium administration with or without a loop diuretic, and the use of demeclocycline or lithium.

## Methods

### Overview

We conducted a systematic review of the medical literature using Medline and Embase. The results from searches of previous studies were processed in the cloud software Covidence (Veritas Health Innovation Ltd). Our objective was to identify and critically evaluate all available studies on urea for hyponatremia resulting from presumed SIADH, including lower-quality evidence such as case series, retrospective, and uncontrolled studies. Although it was not feasible to compare the effect of urea vs a control group (eg, VRAs) via meta-analysis, we pooled the average observed effect of urea on serum sodium, weighted by study size. Lastly, we calculated the sample size for a trial comparing urea with another treatment. We followed the Preferred Reporting Items for Systematic Reviews and Meta-Analyses (PRISMA) reporting guideline. The protocol for the systematic review was preregistered in PROPSERO (CRD42021213569).

### Search Strategy

We conducted our search on 2 databases: the Index Medicus subset within Medline (via the search interface Entrez PubMed) and Embase (via Embase.com). Medline searches also included PubMed Central and related databases. Search terms included combinations of medical subject headings or Emtree terms and other relevant key words (eMethods and eTables 1-4 in Supplement 1). The original search was conducted on October 10, 2019, and we ran a search update on September 7, 2022. Additionally, we conducted a gray search by going through review articles for trials not found through these studies and conducting web searches.

### Inclusion and Exclusion Criteria

We included full-length manuscripts, research letters and brief reports, and abstracts and conference proceedings in English, French, or German in which treatment options studied included oral urea. The patient cohort or subgroup had to consist of only (or had to be close to 100%) patients with hyponatremia and a presumed etiology of SIADH. Any or no comparator were acceptable. End points had to include 1 or more of the following: plasma or serum sodium level; ODS; and resource use, eg, length of stay or costs (or components of either). Identified reviews of urea treatment for hyponatremia were searched for references of original studies. We operationalized the following exclusion criteria for the counts in the PRISMA chart with the short descriptor mentioned in bracket: a manuscript in a language other than English, French, or German (“wrong language”); one of the treatments did not consist of only (or close to 100%) patients with hyponatremia with a presumed etiology of SIADH (“wrong treatment”); a patient cohort or subgroup that did not consist of only (or close to 100%) patients with hyponatremia who had a presumed etiology of SIADH (“wrong patients”); and/or none of the end points match our designated end point (“wrong outcomes”).

### Data Analysis

We extracted characteristics about the studies—such as study type—as well as about the setting, the patient population, the urea dose (as well as comparator treatments, if applicable), baseline sodium and sodium levels after treatment, as well as resources used, such as length of hospital stay, and treatment length. We arbitrarily defined long-term treatment as mean treatment time measured by months. In terms of quantitative synthesis, we attempted a random-effects meta-analysis. However, since there was no one or more uniform treatment available as control group (most studies were uncontrolled), we decided to pool the effects of urea and other treatments, as available, on the sodium level, weighted by sample sizes of the studies. We also calculated the sample size of a potential prospective study that would compare urea with VRAs as well as another one comparing with fluid restriction, assuming a 2-sided α-level of .05 and a power of 80%. Analyses were performed in Excel (Microsoft Corporation) and STATA/SE version 17.0 (Stata Corp).

## Results

In Medline and PubMed, the systematic search identified 330 database entries during the initial run and 29 entries during the update. In Embase, 819 and 278 entries, respectively, were identified. After removing 223 duplicates, there were 1235 records to screen. Of these, 1157 records were excluded based on screening titles and abstracts. For all of the remaining 78 records, full texts were retrieved and evaluated. Removing a further 55 records according to our exclusion criteria left us with 23 studies that fulfilled our inclusion but not our exclusion criteria (Figure).

**Figure.**
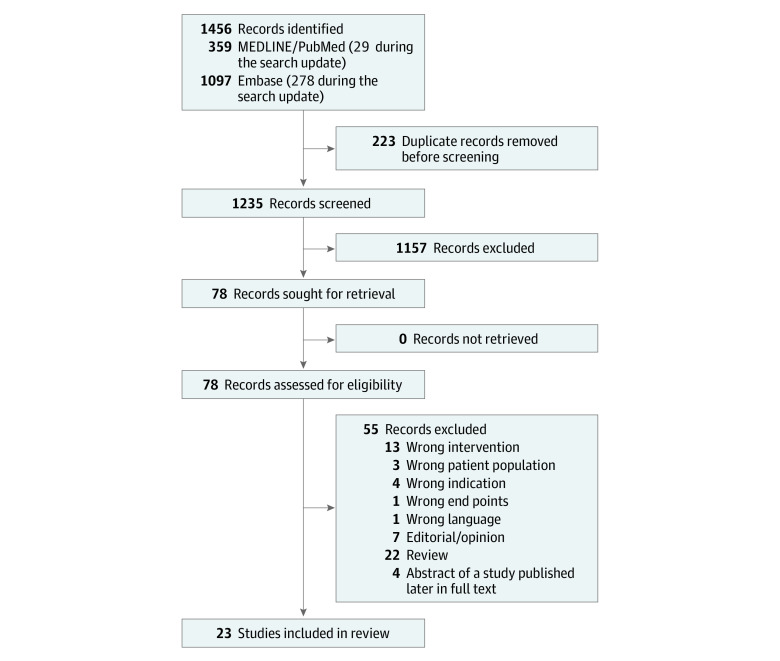
PRISMA Chart

The 23 studies included in our systematic search enrolled 662 participants, of whom 537 had SIADH and 462 had SIADH and were treated with urea (Table 1; eFigures 1-3 in Supplement 1). The median (IQR) number of patients treated with urea was 12 (1-36). There were no randomized clinical trials. Only 2 studies were prospective trials,^5,6^ 20 studies^7,8,9,10,11,12,13,14,15,16,17,18,19,20,21,22,23,24,25,26^ were retrospective trials, and in 1 study^27^ the design was clearly stated. For 8 studies, only abstracts were published.^11,12,17,21,25,27^ All studies investigated orally taken urea. In 3 studies, urea was also given by nasogastric tube.^10,14,20^ Most studies had a recommendation for fluid restriction while treating with urea, in 5 studies fluid restriction was not mentioned^10,11,12,21,27^ and in 5 studies not recommended.^9,14,15,20,25^ Four studies compared urea treatment with vaptans as comparator.^6,11,16,21^ Seven studies (30%) were case reports^7,8,12,17,19,21,22^ of treatment of an SIADH patient with urea. Ten studies reported more than 20 patients with urea treatment for SIADH^9,10,13,14,15,16,18,20,23,27^ with a mean (SD) of 47.1 (20.2) patients.

**Table 1.** Study and Patient Characteristics

Source	No. of participants^a^	Study type	Setting and cohort description	Follow-up time	Baseline sodium, mmol/L	Baseline urine osmolality, mOsmol/kg	Urea dose, g/kg body weight	Primary and secondary end points
Annoni et al,^10^ 2017	40	Retrospective	Belgium; neurosurgical patients with SIADH	0.5 d	133	NR	Mean, 57 g/d	Intracranial pressure
Berkman et al,^24^ 2018	152 (27/5)	Retrospective	Australia (2016); 152 patients with hyponatremia; 27 with SIADH, with 5 receiving urea treatment after fluid restriction failed	15.1 d	Median (IQR) in the euvolemic subgroup, 124 (121-125)	Median (IQR), 331 (234-501) in the euvolemic subgroup	Range, 15-90 g/d	Accuracy of diagnosis, management strategy, change in serum sodium and patient outcomes
Coussement et al,^18^ 2012	24	Retrospective	France (2000-2010); patients with SIADH patients in ICU setting, 18 of 24 patients with a neurological cause	5 d	125 (6)	Mean (SD), 537 (159)	Median, 45 g/d	Time course of serum sodium
Decaux et al,^20^ 2010	85	Retrospective	Belgium; 50 patients in ICU with SIADH with mild hyponatremia, 35 patients with SIADH with severe hyponatremia	2 d	Mild hyponatremia, 128 (4); severe hyponatremia, 111 (3)	Mean (SD), 587 (153)	46 (25) g/d	NR
Decaux et al,^19^ 1980	1	Case report	Belgium (1978); SIADH secondary to cancer	14 d	121	500	Days 1 and 2, 15 g; day ≥3, 30 g	Change in serum sodium
Decaux et al,^23^ 2018	20	Retrospective	Belgium (2011-2016); patients with chronic SIADH	>360 d	Patients receiving 15 g, 127.5 (3.0); patients receiving 30 g, 126 (2)	Patients receiving 15 g, 340; receiving 30 g urea, 595	8 Patients, 15 g; 12 patients, 30 g	Change in serum sodium
Decaux et al,^26^ 1981	7	Retrospective	Belgium (1978-1981); SIADH	7 d	115 (6)	Mean (SD), 514 (79)	30 g/d	NR
Decaux et al,^8^ 1993	1	Case report	Belgium; SIADH	>5 y	120	NR	30 g/d	NR
Gomez-Antunez et al,^25^ 2013	3	Retrospective	Portugal; internal medicine unit	>6 d	Range, 120-129	NR	Range, 15-60 g/d	NR
Gómez Valbuena et al,^21^ 2013	1	Case report	Spain; internal medicine unit	48 d	108	NR	30 g/d	NR
Lindner et al,^22^ 2021	1	Case report	Switzerland (2021); in-hospital setting	11 d	110	412	30-45 g/d	Serum sodium
Lockett et al,^13^ 2019	69	Retrospective	Australia (2015-2017); tertiary care center	3 d	Median (IQR): nadir sodium, 122 (118-126); initial sodium 127 (122-128)	Median (IQR), 551 (422-724)	Unclear	Patients achieving a serum sodium ≥130 mmol/L at 72 h
Maria Belen et al,^11^ 2019	13 (13/6)	Retrospective	Spain (2016); patients with symptomatic SIADH in a tertiary care center: 6 treated with urea, 7 treated with VRA during hospitalization	15 (7-147)	123	NR	Range, 15-30 g/d	Effectiveness and safety of treatments
Mena- Martín et al,^12^ 2020	1	Case report	Spain; outpatients with SIADH secondary to amyotrophic lateral sclerosis	7 d	Nadir sodium, 117; baseline sodium, 127	560	15 g/d	NR
Matthews et al,^17^ 2020	1	Case report	US; SIADH secondary to subdural hemorrhage, postacute care setting	29 d	122	NR	NR	NR
Nervo et al,^15^ 2019	36	Retrospective	Italy (2013-2018); cancer patients with SIADH	60 d	123 (4)	Mean (SD), 452 (174)	Range, 15-60 g/d; mean (SD), 38.8 (14.1) g/d	Mean serum sodium increase after 24 h and prevalence of eunatremia within 14, 30, and 60 d of treatment
Perelló-Camacho et al,^9^ 2022	48	Retrospective	Spain (2015-2021); tertiary care center	2.95 (6.29) mo	Nadir sodium, 119.8 (5.0); baseline sodium, 125.6 (4.1)	Mean (SD), Mean (SD), 395.7 (128.5)	Range, 15-45 g/d	Pre-posttreatment serum sodium
Perez et al,^27^ 2020	33	Unclear	Spain (2019); tertiary care center	9 mo	125 (4)	NR	NR	Serum sodium level before treatment with urea at 24 h, 48 h, 14 d, and 60 d
Pierrakos et al,^14^ 2012	42	Retrospective	Belgium (2003-2008); SIADH secondary to spontaneous subarachnoid hemorrhage in an ICU	NR	127 (2)	Mean (SD), 498 (125)	50 g/d (range, 40-60 g/d)	NR
Rondon-Berrios et al,^16^ 2018	58 (58/12)	Retrospective	US (2016-2017); subgroup of 12 patients who received urea as the sole drug therapy	Median (IQR), 4.5 d (3-8 d)	Patients treated with urea only, 125 (122-127)	Median (IQR), 365 (361-561)	7.5-90 g/d	Changes in plasma sodium at 24 h and the end of therapy as well as the proportion of patients who achieved plasma sodium ≥135 meq/L
Sejpal et al,^7^ 2022	1	Case report	US; SIADH secondary to neuroborreliosis	NR	122	627	15 g/d for 5 d	NR
Soupart et al,^6^ 2012	13	Prospective long-term study	Belgium (2007-2009); prospective, long-term study in patients with chronic SIADH	2 y	Before VRA, 125 (3); before urea, 126 (5)	Mean (SD), 487 (162)	15-30 g/d	Serum sodium level
Woudstra et al,^5^ 2020	13	Prospective case series	Netherlands (2017-2019); hospitalized patients with SIADH	Median (IQR), 5 (2-10) d	124 (range, 122-128)	Median (IQR), 553 (478.5-743)	Range, 0.25-0.50 g/kg/d, 1 daily dose except for doses >40 g/d	Proportion of patients reaching normonatremia

Abbreviations: ICU, intensive care unit; NR, not reported; SIADH, syndrome of inappropriate secretion of antidiuretic hormone; VRA, vaptan receptor antagonist.

^a^ The numbers in brackets refer to the subgroups of patients, if applicable, with SIADH (first number) and with SIADH treated with urea (second number).

The majority of studies included patients with a mean baseline serum sodium of 120 mmol/L or higher or below 130 mmol/L at the start of urea treatment (Table 1; eFigure 4 in Supplement 1). Three studies analyzed patients with serum sodium concentration below 120 mmol/L at baseline^21,22,28^ with 2 studies including patients with serum sodium baseline concentrations 110 mmol/L or lower.^21,22^ One study included patients with SIADH who had mild hyponatremia with a mean serum sodium concentration at treatment start of 133 mmol/L.^10^ The pooled mean baseline sodium was 125.0 (95% CI, 122.6-127.5) mmol/L.

All studies reported treatment effects of urea with the main outcome being increase in serum sodium or percentage of sodium normalization (Table 2; eFigures 5 and 6 in Supplement 1). The median (IQR) treatment duration, weighted by sample size, was 5 (2-90) days. Five studies investigated long-term treatment effects of urea in SIADH.^6,9,15,23,27^ Urea, again weighed by sample size, increased the serum sodium by a mean of 9.6 mmol/L (95% CI, 7.5-11.7 mmol/L) whereas VRAs increased it by 10.5 mmol/L (95% CI, 7.6-13.3 mmol/L) and fluid restriction by 7.8 mmol/L (95% CI, −9.8 to 25.5 mmol/L). There was only 1 study that reported the course of serum sodium with no treatment,^24^ and the change in serum sodium was zero. The first significant change in serum sodium with urea occurred within a median of 1 day. The mean increase in serum sodium after the first 24 hours was 4.9 mmol/L (95% CI, 0.5-9.3 mmol/L).

**Table 2.** Efficacy and Safety Results

Source	Baseline and/or nadir sodium, mmol/L	Final sodium, mmol/L	First significant and maximum increase	Sodium change/d	Other efficacy results	Safety or adverse events	Length of stay; resource use	Study conclusion
Annoni et al,^10^ 2017	133	141	First day	>12 h, 8	Median intracranial pressure decrease, 14 to 11 mm Hg	NR	NR	Patients with acute brain injury and hyponatremia treated with urea had significant decrease in intracranial pressure, independent of changes in plasma sodium concentrations
Berkman et al,^24^ 2018	Median (IQR): urea, 124 (120-125); fluid restriction, 123 (119-125)	Median: urea, 13.5; fluid restriction, 7; no treatment, 0	24-48 h (day 2)	0-24 h: urea, 2.5; fluid restriction, 2.5; 24-48 h: urea, 4; fluid restriction, 2-5; 38-72 h: urea, 3; fluid restriction, 0	NR	No ODS; serious adverse events (3/5 patients) included unpleasant taste, nausea, and reflux	NR	Treatment associated with improvement in serum sodium for nonresponsive or restricted patients; hypertonic (3%) saline considered treatment of choice for severe symptomatic hyponatraemia but with careful monitoring
Coussement et al,^18^ 2012	125 (6)	136.2 (4.1) (fourth day of treatment)	2 d (second day of treatment: mean [SD] sodium, 131.4 [3.5] mmol/L)	2 d, 6.6 mmol/L; 4 d, 11.4 mmol/L	NR	NR	NR	Urea administration appears useful for the treatment of SIADH-associated hyponatremia in critically ill patients
Decaux et al,^20^ 2010	Mild group, 128 (4); severe group, 111 (3)	Mild, 136 (5); severe, 122 (4)	Mild, 2 d; severe, 1 d	4; 11	Hyponatremia recurred in 6 patients with moderate hyponatremia when urea was stopped	Mild: 7 patients developed hypernatremia (maximum, 155); severe: serum sodium increased >12 mmol/L after 1 d in 12 patients and >18mmol/L/48 h in 13 patients; no ODS developed; 7 patients developed hypokalemia (range, 2.3-3.4 mmol/L)	Mean total urea duration, 6 d (range, 2-42 d)	Urea is a simple and inexpensive therapy to treat euvolemic hyponatremia in the ICU
Decaux et al,^19^ 1980	121	133	NR	Day 1, 5 mmol/L; day 2, 1 mmol/L; day 3, 3 mmol/L	NR	NR	NR	Urea is a valid treatment of SIADH, particularly among patients with acute hyponatremia
Decaux et al,^23^ 2018	128 (3) in 8 patients with 15 g urea; 126 (2) in 12 patients with 30 g urea	136 (1) in 8 patients with 15 g urea; 136 (2) in 12 patients with 30 g urea	NR	NR	All patients normalized their serum sodium with urea and mild water restriction	Gustative intolerance in 2 patients, continued urea with addition of NaHCO_3_, sucrose and citric acid to urea solution, no case of hypernatremia reported	NR	Urine osmolality is accurate estimating the response to mild water restriction, acceptable in the long-term; however, small doses of urea could likely patients with low solute intake and high urine osmolality.
Decaux et al,^26^ 1981	115 (6)	136 (3.5)	NR	NR	NR	NR	NR	Urea is a safe and efficacious treatment for SIADH
Decaux et al,^8^ 1993	120	>130	NR	NR	NR	NR	NR	Oral urea, even during long periods, is a safe and effective therapeutic approach for patients with chronic SIADH, not controlled
Gomez-Antunez et al,^25^ 2013	Range: 120-129	>130 Total patients after 6 (5-7) days of treatment	NR	NR	All patients responded to treatment	Increase in blood urea levels in 1 patient	NR	Urea is an efficient and safe method to manage hyponatremia that can be well tolerated and can help avoid fluid restriction
Gómez Valbuena et al,^21^ 2013	108	Urea, 134; tolvaptan, 130, fluid restriction, 120	NR	No parallel groups, 1 patient, consecutively treatment with tolvaptan and then urea; change in sodium, 4.6 mmol/L	NR	NR	Daily costs €33.5-€69 per d with tolvaptan and €0.30 per d for urea costs	Use of urea as alternative treatment therapy tolvaptan in SIADH can get the same result that tolvaptan in normalization of serum sodium, a markedly lower cost
Lindner et al,^22^ 2021	110	133 (At discharge); 139 (5 d after discharge with continuing urea treatment)	NR	NR	NR	0	6 d	Vaccination against SARS-CoV-2 triggered SIADH
Lockett et al,^13^ 2019	Median (IQR): initial, 127 (122-128); nadir, 122 (118- 126)	NR	NR	NR	Fluid restriction as first-line treatment, 65.4%; urea as first-line treatment, 21.8% and second line in 78.2%	22.7% With distaste; 0 overcorrections	NR	Urea is safe and effective in fluid restriction-refractory hyponatraemia; recommended starting dose of urea ≥30 g/d in patients with SIADH and moderate to profound hyponatraemia unable to undergo or have failed fluid restriction
Maria Belen et al,^11^ 2019	123	Urea group, 133; tolvaptan group, 133	NR	Mean (SD) increase in sodium in 24 h: urea, 4.57 (0-8); 9.9 (−3 to 21) in tolvaptan (in 3 cases >12 mEq/L in 24 h)	3 Patients with urea achieved eunatremia, 2 patients died; tolvaptan group, 3 patients eunatremic, 3 deaths (due to complications of the underlying diseases)	3 Patients in tolvaptan group with change in sodium >12 within 1 d	NR	In SIADH, tolvaptan has shown to be moderately effective, but the correction was too rapid; urea is an alternative of moderate efficacy but safer, allowing its ambulatory use
Mena- Martín et al,^12^ 2020	Nadir, 117; baseline at start of treatment, 127	136 At day 7 of treatment	NR	NR	NR	NR	Outpatient	In patients with SIADH, urea administration increases the urinary excretion of solutes and water, significantly raising sodium levels
Matthews et al,^17^ 2020	122	133 (Day 4 after treatment with urea)	Sodium after 48 h, 128 mmol/L	NR	NR	NR	NR	Dangerously low sodium level successfully treated with urea; during treatment, the patient was able to continue his rehabilitation amidst an intricate medical course
Nervo et al,^15^ 2019	123 (4)	128 (4) after 24 h	Sodium increase in 24 h, 5 (3) mmol/L	NR	55.6% With eunatremia after 14 d of treatment; 86.1% after 30 d; 91.7% after 60 d; no overcorrection in any patient	10 Patients interrupted urea treatment, 5 because of rapid tumor progression, 5 because of drug taste	NR	Urea was effective in correcting chronic hyponatremia among cancer patients with SIADH; almost all patients reached eunatremia within 1 mo, and was well tolerated
Perelló-Camacho et al,^9^ 2022	Nadir, 119.8 (5.0); treatment start, 125.6 (4.1)	134.4 (4.9)	Sodium levels: day 1, 129 mmol/L; day 7, 133.3 (4.9)	NR	NR	Mild digestive symptomatology, 2 patients; refused based on palatability, 2 patients	Cost reduction, 87.9% vs treatment with tolvaptan	Urea shown to be safe and cost-effective treatment option for hyponatremia caused by SIADH
Perez et al,^27^ 2020	125 (4)	After 14 d and 60 d, 134 (4)	24 h, 127 (5); 48 h, 129 (5)	NR	NR	NR	NR	No significant differences in plasma sodium values before and after urea treatment
Pierrakos et al,^14^ 2012	127 (2)	Total NR; all patients became eunatremic at median 3 d of urea treatment	3 mmol/L (1-6) increase in <24 h	3 mmol/L (1-6) increase in <24h	Hyponatremia reversed in all patients, plasma sodium returning to >130 and 135 mEq/L after a median (IQR) 1 and 3 d, respectively; median (IQR) sodium increase: day 1, 3 mEq/L (1-6); day 2, 5 (3-10) mEq/L	No gastrointestinal or hemodynamic adverse effects; sodium increased ≥12 mEq/L during the first day of therapy in 4 patients, withou clinical deterioration; 2 patients developed transient hypernatremia (maximum, 149 mEq/L), which resolved ≤24 h	NR	Urea effective for SIADH-related hyponatremia in patients with SAH; urea generally well tolerated
Rondon-Berrios et al,^16^ 2018	Median (IQR) urea-only patients, 125 (122-127)	Median (IQR), 131 (129-136)	Median (IQR) <24 h, 2.5 (0- 4.5)	Median (IQR) sodium for urea-only patients in 24 h: 2.5 mEq/L (0-4.5) vs untreated patients, −0.5 mEq/L (−2.5 to 1.5); *P* = .04)	No difference in change in plasma sodium between urea-only treated patients and urea-untreated patients (median [IQR], 6 mEq/L [3.5-10] vs 5.5 mEq/L [3-7.5]; *P* = .51); a greater proportion of urea only treated patients achieved normal sodium at the end of therapy compared with urea-untreated patients (33% vs 8%; *P* = .08)	2% Discontinued urea, reportedly due to dysgeusia	Urea-only treated patients vs urea-untreated patients: median (IQR) length of hospital stay, urea-treated, 6 d [3.5-7] vs untreated, 6 d [4-6] (*P* = .74)	Urea effective and safe for the treatment of inpatient hyponatremia, and well tolerated
Sejpal et al,^7^ 2022	122	139	NR	NR	NR	NR	NR	SIADH resolves with successful treatment of the underlying Lyme infection as was seen in our case
Soupart et al,^6^ 2012	Before vaptan, 125 (3); before urea, 126 (5)	After 1 y: vaptan group, 135 (3); urea group, 135 (2)	NR	NR	NR	Hypernatremia in 1 urea patient without complications	NR	Urea has efficacy similar to that of vaptans for treatment of chronic SIADH
Woudstra et al,^5^ 2020	124	Median (IQR), 135 (130-137)	NR	Day 1, 128 (123-130); day 2, 130 (127-132); end of inpatient treatment, 135 (130-137)	Normonatremia in 8/13 patients (62%) at the end of in-hospital treatment; median serum sodium level at 2 wk follow-up was 134 mmol/L (range, 132-141); median serum sodium level at 6-8 wk, 134 mmol/L (range, 128-140)	Urea was discontinued due to nausea after 4 d of therapy in 1 patient, 6 patients reported light to moderate intake difficulties due to the taste of urea; urea was not discontinued in these patients; no overcorrection was observed	NR	Urea is an effective second-line treatment strategy for hospitalized patients with hyponatraemia due to SIADH; distaste and nausea were reported adverse effects in population, but mostly did not lead to early discontinuation

Abbreviations: ICU, intensive care unit; NR, not reported; ODS, osmotic demyelination syndrome; SAH, subarachnoid hemorrhage; SIADH, syndrome of inappropriate antidiuretic hormone secretion.

Urea was well tolerated with only a few studies reporting adverse events, mainly consisting of distaste and or dysgeusia. Additionally, nausea, gastroesophageal reflux, and hypernatremia were reported in few cases. In a small subset of patients, gustative intolerance led to refusal to take urea or to interruption of drug intake.^5,9,15,16,23^ There were no cases of ODS in SIADH patients with urea treatment. Only 1 study reported overcorrection of serum sodium (an increase of more than 12 mmol/L in 24 hours) with urea treatment without clinical deterioration.^14^ In this study patients with nontraumatic subarachnoid hemorrhage with mild hyponatremia (mean [SD], 127 [2] mmol/L) were treated with a mean dose of 50 g urea, mainly via nasogastric tube.

The sample size for a noninferiority trial comparing urea and VRAs could be quite small, maybe under 10 patients depending on the safety margin. A trial between urea and fluid restriction with a superiority design would require at least 106 patients if patients were equally distributed between the 2 groups.

## Discussion

We systematically searched previous studies including patients with SIADH who received urea treatment. In the 23 studies analyzed, 537 patients with SIADH were reported and 462 were treated with urea as the only therapy. Due to the absence of randomized clinical trials, in particular ones with adequate sample sizes, there is no definite evidence for the efficiency of urea in the treatment of hyponatremia in patients with SIADH. However, the available, lower-quality evidence suggests that urea is an effective treatment option with a very low risk of overcorrection and rarely reported adverse effects aside from dysgeusia.

In the pooled analysis we present here, urea increased serum sodium by 10.2 mmol/L within the reported follow-up time and seemed to be as effective in increasing serum sodium concentration as the VRA tolvaptan (serum sodium increase of 10.4 mmol/L until end of follow up). The rise in serum sodium starts as early as 24 to 48 hours after treatment initiation. Nearly all reported patients improved their serum sodium concentration with urea treatment to a level above 130 mmol/L. The average urea dose in the 23 studies was 15 g to 30 g urea per day with no reported adjustment to urine osmolality. For context, the dose recommendation in the European guidelines for treatment of SIADH^4^ with urea is 0.25 to 0.50 g/kg per day. Urea raises serum sodium by increasing free water excretion through an osmotic-diuretic mechanism. The amount of free water excretion and therefore effectiveness of urea depends among others on the applied dose of urea and the urine osmolality. With increasing urine osmolality, the necessary dose of urea to improve serum sodium is higher. A practical guideline on dosing of urea in hyponatremia patients with regard to the initial urine osmolality exists.^29^ The risk of overcorrection of serum sodium concentration with urea is very low. Overcorrection is assumed to be a major contributor to the development of ODS even though a recent work showed only very low incidences of ODS in a large retrospective cohort of patients with hyponatremia and found no correlation between rapid corrections of serum sodium and ODS.^30^ Of note, the incidence was higher in patients with serum sodium below 110 mmol/L and overcorrection should, in our opinion, still be avoided especially in high-risk patients. The risk for ODS is still a matter of concern, and especially in patients at higher risk urea might be a safe and effective therapeutic option. However, in animal models, urea seemed to protect from ODS.^31,32^ Regarding the most common adverse effect of urea, dysgeusia, previous studies suggest that taste of urea can be improved by adding sucrose and citric acid^23^ or by dissolving urea in sweetened beverages.

In case of severe acute hyponatremia, 3% sodium chloride infusions will most likely remain standard of care because they have an even more rapid onset of effect and are easy to titrate. However, in the outpatient setting, in hospitalized patients with less severe and less acute hyponatremia, and in patients with triggers that are not easy to control such as psychotropic medications, urea could be a safe and effective treatment option at low costs. Importantly, a dose-effect relationship of urea on free water excretion may be present which is dependent on urinary osmolality. Randomized clinical trials against the criterion standard of 3% sodium chloride infusions in acute hyponatremia from SIADH or VRA in chronic SIADH would be needed to further evaluate to potential role of urea in the treatment of hyponatremia in SIADH.

### Limitations

There were several limitations to this systematic review. First, evidence from randomized clinical trials does not exist and few studies even have control groups. In addition to confounding by indication for urea therapy, it is very likely that publication bias was also present since we only identified trials with positive treatment effects. Also, there was inconsistent handling of fluid restriction. Finally, treatment of the trigger situation (eg, infection, pain, brain trauma) itself can improve serum sodium concentration in SIADH patients and in uncontrolled trials this effect cannot be separated.

## Conclusions

In this systematic review, there was no definite evidence regarding urea as a treatment for SIADH. Randomized clinical trials would be necessary to prove the efficacy of urea for this indication. However, the data identified in this systematic review support the hypothesis that urea might be an effective, safe, and inexpensive treatment option for patients with SIADH.

## Notes

Supplement 1.
eMethods.
**eTable 1.** Original Search Strategy in Medline **eTable 2.** Original Search Strategy in Embase **eTable 3.** Search Strategy for the Update in Medline **eTable 4.** Search Strategy for the Update in Embase **eFigure 1.** Bar Chart of the Number of Subjects **eFigure 2.** Histogram of the Number of Subjects **eFigure 3.** Box and Whisker Plot of the Follow-up Time **eFigure 4.** Box and Whisker Plot of the Baseline Sodium **eFigure 5.** Box and Whisker Plot of the Final Sodium in the Urea Group **eFigure 6.** Box and Whisker Plot of the Change (Delta) in Sodium

Supplement 2.
Data Sharing Statement
