# Supplementary material for: Use of Urea for the Syndrome of Inappropriate Secretion of Antidiuretic Hormone: A Systematic Review
Source: JAMA Netw Open. 2023 Oct 30;6(10):e2340313. doi: 10.1001/jamanetworkopen.2023.40313 (PMC10616719; doi:10.1001/jamanetworkopen.2023.40313)
Supplement: Supplement 1. — eMethods. eTable 1. Original Search Strategy in Medline eTable 2. Original Search Strategy in Embase eTable 3. Search Strategy for the Update in Medline eTable 4. Search Strategy for the Update in Embase eFigure 1. Bar Chart of the Number of Subjects eFigure 2. Histogram of the Number of Subjects eFigure 3. Box and Whisker Plot of the Follow-up Time eFigure 4. Box and Whisker Plot of the Baseline Sodium eFigure 5. Box and Whisker Plot of the Final Sodium in the Urea Group eFigure 6. Box and Whisker Plot of the Change (Delta) in Sodium [file jamanetwopen-e2340313-s001.pdf]

Supplemental Online Content

Wendt R, Fenves AZ, Geisler BP. Use of urea for the syndrome of inappropriate secretion of antidiuretic hormone. *JAMA Netw Open.* 2023;6(10):e2340313. doi:10.1001/jamanetworkopen.2023.40313

eMethods.
eTable 1. Original Search Strategy in Medline
eTable 2. Original Search Strategy in Embase
eTable 3. Search Strategy for the Update in Medline
eTable 4. Search Strategy for the Update in Embase
eFigure 1. Bar Chart of the Number of Subjects
eFigure 2. Histogram of the Number of Subjects
eFigure 3. Box and Whisker Plot of the Follow-up Time
eFigure 4. Box and Whisker Plot of the Baseline Sodium
eFigure 5. Box and Whisker Plot of the Final Sodium in the Urea Group
eFigure 6. Box and Whisker Plot of the Change (Delta) in Sodium

This supplemental material has been provided by the authors to give readers additional information about their work.

© 2023 Wendt R et al. *JAMA Network Open.*

eMethods.

Search Strategy – Original Search

Medline

- **Databases:** PubMed/Medline/Pre-Medline 1946 to October 2019
- **Limits/Filters**:
    - Species: not animal or mixed study
    - Language: English, French, or German
- **Search Date:** October 10, 2019
- **Search Terms and Strategy:**

Embase

- **Database:** Embase Classic + Embase 1967 (plus <1966) to October2019
- **Limits/Filters:**
    - Species: Humans
    - Language: English, French, or German
- **Search Date:** October 10, 2019
- **Search Terms and Strategy:**

Search Strategy – Update

Medline

- **Databases:** PubMed/Medline/Pre-Medline October 2019 to September 2022
- **Limits/Filters**:
    - Species: not animal or mixed study
    - Language: English, French, or German
    - Date 2019/10/10 to 2023/12/31
- **Search Date:** September 7, 2022
- **Search Terms and Strategy:**

Embase

- **Database:** Embase Classic + Embase 2019 to September 2022
- **Limits/Filters:**
    - Species: Humans
    - Language: English, French, or German
- **Search Date:** September 7, 2022
- **Search Terms and Strategy:**

© 2023 Wendt R et al. *JAMA Network Open.*

eTable 1: Original Search Strategy in Medline.

#	Searches	Results
1	"Urea"[Mesh] OR urea[tiab] NOT ("Blood Urea Nitrogen"[Mesh] OR "Blood Urea Nitrogen"[tiab])	60,571
2	"Inappropriate ADH Syndrome"[Mesh] or "syndrome of inappropriate antidiuretic hormone secretion"[tiab] "syndrome of inappropriate ADH secretion"[tiab] OR SIADH[tiab]	1,110
3	#1 AND #2	61
4	"Hyponatremia"[Mesh] OR Hyponatremia[tiab] OR hyponatraemia[tiab]	10,458
5	#2 OR #4	10,806
6	#1 AND #5	**330**

eTable 2: Original Search Strategy in Embase.

#	Searches	Results
1	'urea'	61,714
2	'urea nitrogen blood level' OR 'blood urea nitrogen creatinine ratio' OR 'blood urea nitrogen test kit'	17,647
3	'urea derivative'	1,043
4	#2 OR #3	18,676
5	#1 NOT #4	43,038
6	'inappropriate vasopressin secretion'/exp OR 'inappropriate vasopressin secretion' OR 'inappropriate adh syndrome' OR 'syndrome of inappropriate antidiuretic hormone secretion' OR 'syndrome of inappropriate adh secretion' OR 'siadh'	3,969
7	#5 AND #6	244
8	'hyponatremia' OR 'hyponatraemia'	24,596
9	#6 OR #8	25,837
10	#5 AND #9	**819**

© 2023 Wendt R et al. *JAMA Network Open.*

eTable 3: Search Strategy for the Update in Medline.

#	Searches	Results
1	"Urea"[Mesh] OR urea[tiab] NOT ("Blood Urea Nitrogen"[Mesh] OR "Blood Urea Nitrogen"[tiab])	6,508
2	"Inappropriate ADH Syndrome"[Mesh] or "syndrome of inappropriate antidiuretic hormone secretion"[tiab] "syndrome of inappropriate ADH secretion"[tiab] OR SIADH[tiab]	150
3	#1 AND #2	6
4	"Hyponatremia"[Mesh] OR Hyponatremia[tiab] OR hyponatraemia[tiab]	1,312
5	#2 OR #4	1,341
6	#1 AND #5	**29**

© 2023 Wendt R et al. *JAMA Network Open.*

eTable 4: Search Strategy for the Update in Embase.

#	Searches	Results
1	'urea' AND ([embase]/lim OR [embase classic]/lim) AND [2019-2022]/py AND [humans]/lim AND ([english]/lim OR [french]/lim OR [german]/lim)	21,546
2	('urea nitrogen blood level' OR 'blood urea nitrogen creatinine ratio' OR 'blood urea nitrogen test kit') AND ([embase]/lim OR [embase classic]/lim) AND [2019-2022]/py AND [humans]/lim AND ([english]/lim OR [french]/lim OR [german]/lim)	9,405
3	'urea derivative' AND ([embase]/lim OR [embase classic]/lim) AND [2019-2022]/py AND [humans]/lim AND ([english]/lim OR [french]/lim OR [german]/lim)	180
4	#2 OR #3	9,585
5	#1 NOT #4	11,961
6	('inappropriate vasopressin secretion'/exp OR 'inappropriate vasopressin secretion' OR 'inappropriate adh syndrome' OR 'syndrome of inappropriate antidiuretic hormone secretion' OR 'syndrome of inappropriate adh secretion' OR 'siadh') AND ([embase]/lim OR [embase classic]/lim) AND [2019-2022]/py AND [humans]/lim AND ([english]/lim OR [french]/lim OR [german]/lim)	913
7	#5 AND #6	77
8	('hyponatremia' OR 'hyponatraemia') AND ([embase]/lim OR [embase classic]/lim) AND [2019-2022]/py AND [humans]/lim AND ([english]/lim OR [french]/lim OR [german]/lim)	7,892
9	#6 OR #8	8,152
10	#5 AND #9	**278**

© 2023 Wendt R et al. *JAMA Network Open.*

Quantitative Summary of the Identified Studies

Number of Subjects

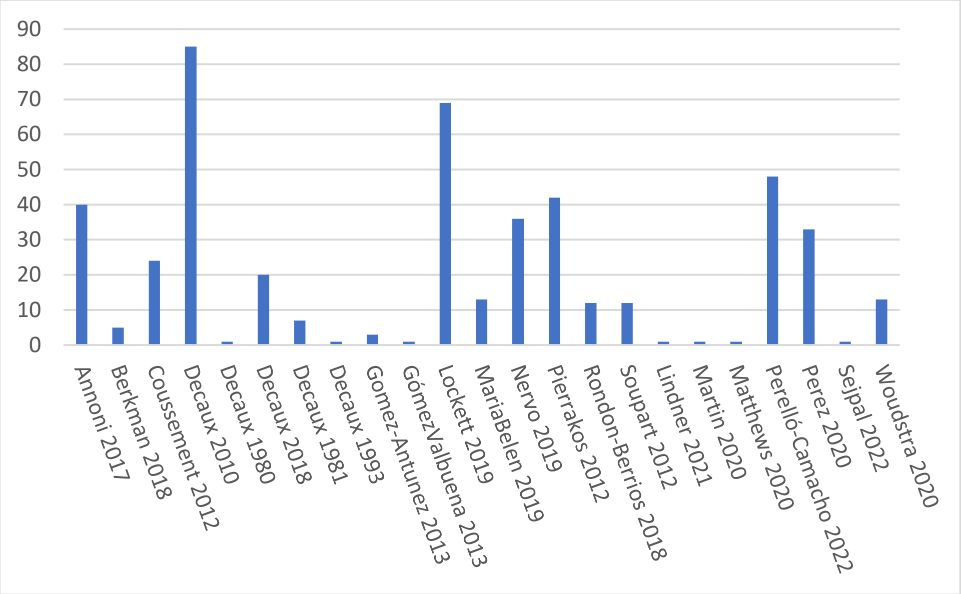

eFigure 1: Bar Chart of the Number of Subjects.

Median: 12
Interquartile range interquartile range (IQR): 1; 36
Range: 1; 152

© 2023 Wendt R et al. *JAMA Network Open.*

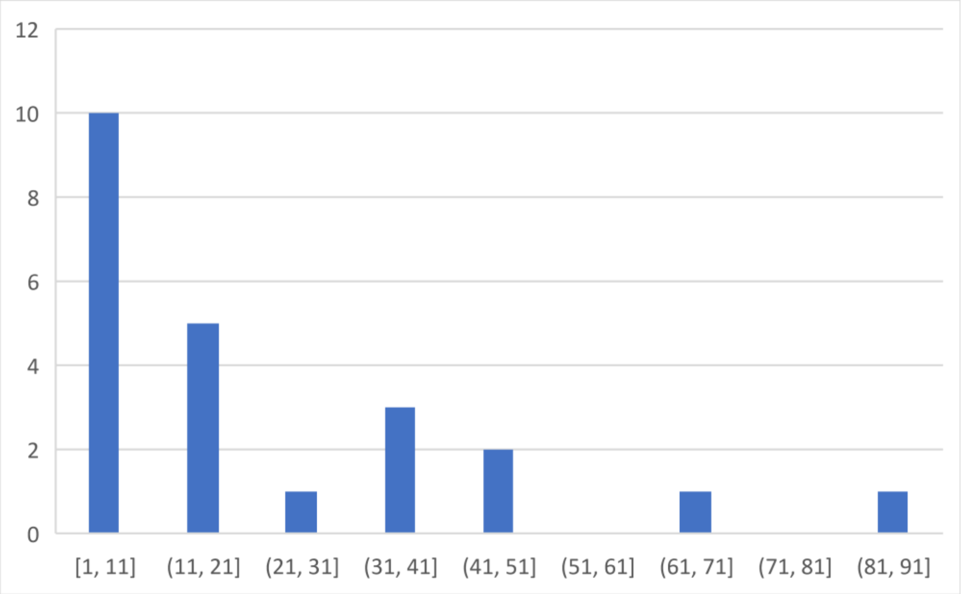

eFigure 2: Histogram of the Number of Subjects.

© 2023 Wendt R et al. *JAMA Network Open.*

Follow-up Time

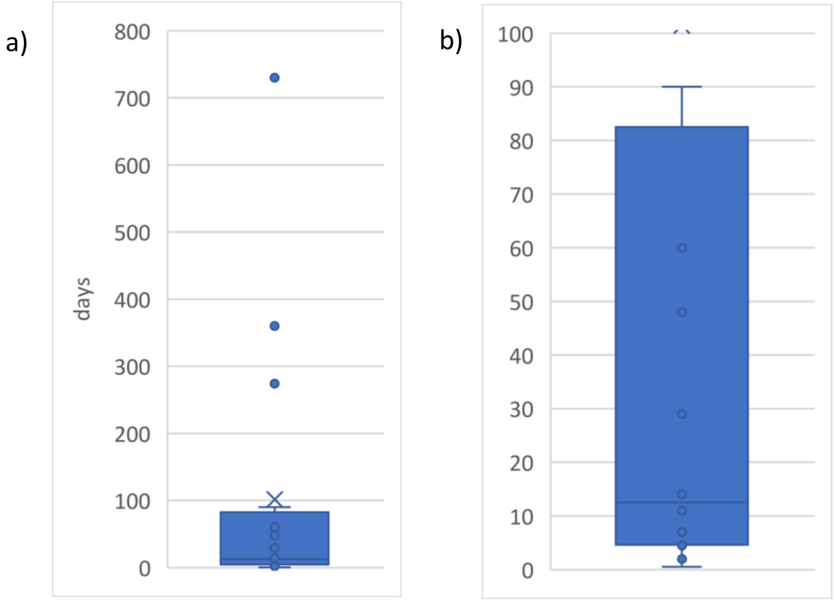

eFigure 3: Box- and Whisker Plot of the Follow-up Time. a) entire range; b) close up of the range 0-100 days.

Median (weighted by sample sizes): 5 (IQR: 2; 90) days

Mean (weighted by sample sizes): 77.7 (95% confidence interval [CI]: 2.5; 152.8) days

© 2023 Wendt R et al. *JAMA Network Open.*

Baseline Sodium

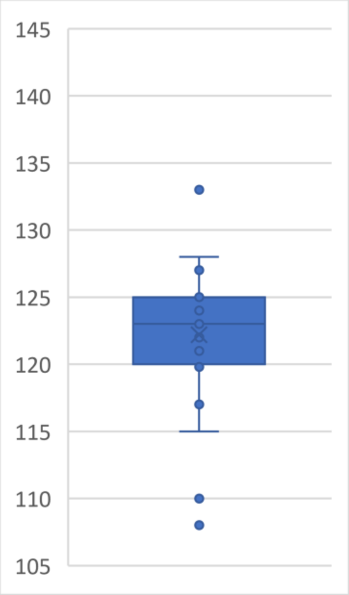

eFigure 4: Box- and Whisker Plot of the Baseline Sodium.

Mean (weighted by sample sizes): 125.0 (95% CI: 122.6; 127.5) mmol/L

© 2023 Wendt R et al. *JAMA Network Open.*

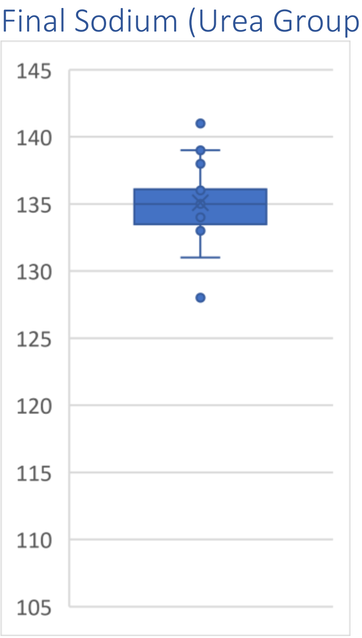

Mean (weighted by sample sizes): 135.0

95% CI: 133.0; 137.0

eFigure 5: Box- and Whisker Plot of the Final Sodium in the Urea Group.

© 2023 Wendt R et al. *JAMA Network Open.*

Delta Sodium

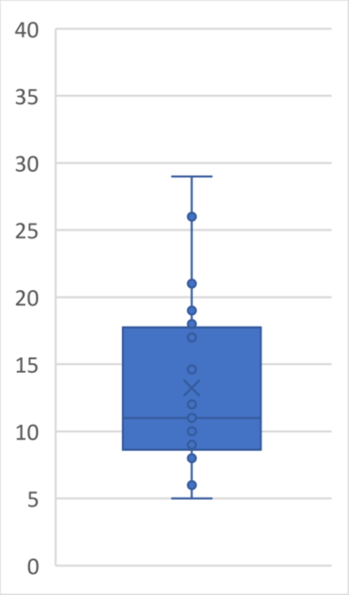

eFigure 6: Box- and Whisker Plot of the Change (Delta) in Sodium.

Delta sodium by subgroup, weighted by sample size:

- For the urea group: mean 9.6 (95% CI: 7.5; 11,7) mmol/L
- For the vaptan group: mean 10.5 (95% CI: 7.6; 13.3) mmol/L
- Fluid restriction group: 7.8 (95% CI: -9.8; 25.5) mmol/L
- No treatment: nil

First Day of Sodium Change After Urea

Median: 1 (IQR: 1; 2) day

Delta Sodium After First Day of Urea

Mean: 4.9 (95% CI: 0.5; 9.3) mmol/L

© 2023 Wendt R et al. *JAMA Network Open.*
