# Supplementary material for: Use of Urea for the Syndrome of Inappropriate Secretion of Antidiuretic Hormone: A Systematic Review
Source: JAMA Netw Open. 2023 Oct 30;6(10):e2340313. doi: 10.1001/jamanetworkopen.2023.40313 (PMC10616719; doi:10.1001/jamanetworkopen.2023.40313)
Supplement: Supplement 2. — Data Sharing Statement [file jamanetwopen-e2340313-s002.pdf]

Data Sharing Statement

Wendt. Use of Urea for the Syndrome of Inappropriate Secretion of Antidiuretic Hormone. *JAMA Netw Open*. Published October 30, 2023. doi:10.1001/jamanetworkopen.2023.40313

Data
Data available: Yes
Data types: Data (not involving human participants)
How to access data: Data will be provided upon reasonable request to Ralph.Wendt@SanktGeorg.DE
When available: beginning date: 07-13-2023

Supporting Documents
Document types: None

Additional Information
Who can access the data: Data will be provided upon reasonable request to all parties.
Types of analyses: Data will be provided upon reasonable request for all types of analyses.
Mechanisms of data availability: Data will be provided upon reasonable request with or without investigator support; no proposal or data access agreement will be necessary.
